# Advances in tissue engineering for the repair of growth plate injuries

**DOI:** 10.3389/fbioe.2025.1608923

**Published:** 2025-09-16

**Authors:** Wenla Wang, Wenxiang Zeng, Qingyu Tu, Qing Li, Jindi Xu, Wei Zhuang

**Affiliations:** ^1^ Research Institute of Orthopedics, Jiangnan Hospital Affiliated to Zhejiang Chinese Medical University, Hangzhou, China; ^2^ Hangzhou Xiaoshan Hospital of Traditional Chinese Medicine, Hangzhou, China

**Keywords:** growth plate injury, tissue engineering, seed cells, growth factors, scaffold material, 3D printing technology

## Abstract

The growth plate is a cartilage tissue located between the epiphysis and diaphysis of long bones, responsible for the longitudinal growth of the skeleton. Due to its limited regenerative capacity, when the growth plate is damaged, it is typically replaced by inappropriate bone tissue, leading to the formation of bony bridges. These bony bridges not only restrict normal skeletal growth but may also cause limb length discrepancies, angular deformities, and functional impairments. Although traditional clinical treatments have shown some effectiveness, they are often associated with severe complications and poor prognoses. Therefore, the development of effective therapeutic strategies to prevent the formation of bony bridges and promote the repair and regeneration of the growth plate has become a current research focus. Cartilage tissue engineering, as an emerging therapeutic approach, restore the function of the growth plate through the substitution or repair of damaged cartilage tissue, has been widely applied in the repair of growth plate injuries. Cartilage tissue engineering for growth plate injury primarily relies on three key components: seed cells, growth factors, and scaffold materials. Seed cells provide the basis for cartilage regeneration, typically using autologous or allogeneic chondrocytes, mesenchymal stem cells, etc.,; growth factors such as bone morphogenetic proteins (BMPs) and transforming growth factor-beta (TGF-β) promote cell proliferation and differentiation, while regulating the synthesis of cartilage matrix; scaffold materials provide three-dimensional structural support, offering a platform for directed cell growth and tissue repair. In recent years, with continuous advancements in biomaterials and innovations in tissue engineering techniques, cartilage tissue engineering has shown promising prospects for application. This article systematically reviews the latest research progress on cartilage tissue engineering in the repair of growth plate injuries, based on a comprehensive search and analysis of relevant literature from databases such as PubMed and CNKI. The paper focuses on the classification and stages of growth plate injuries and discusses the three essential elements of tissue engineering treatment for growth plate injury.

## 1 Introduction

Growth plate, also known as the epiphyseal plate, is a thin layer of cartilage tissue located between the epiphysis and metaphysis of long bones. In immature skeletal systems, growth plate serves as the primary site of growth and development for long bones, facilitating longitudinal growth through endochondral ossification (Racine and Serrat, 2020; Ağırdil, 2020). Growth plate primarily consists of chondrocytes and extracellular matrix (Matsushita et al., 2020).

**FIGURE 1:**
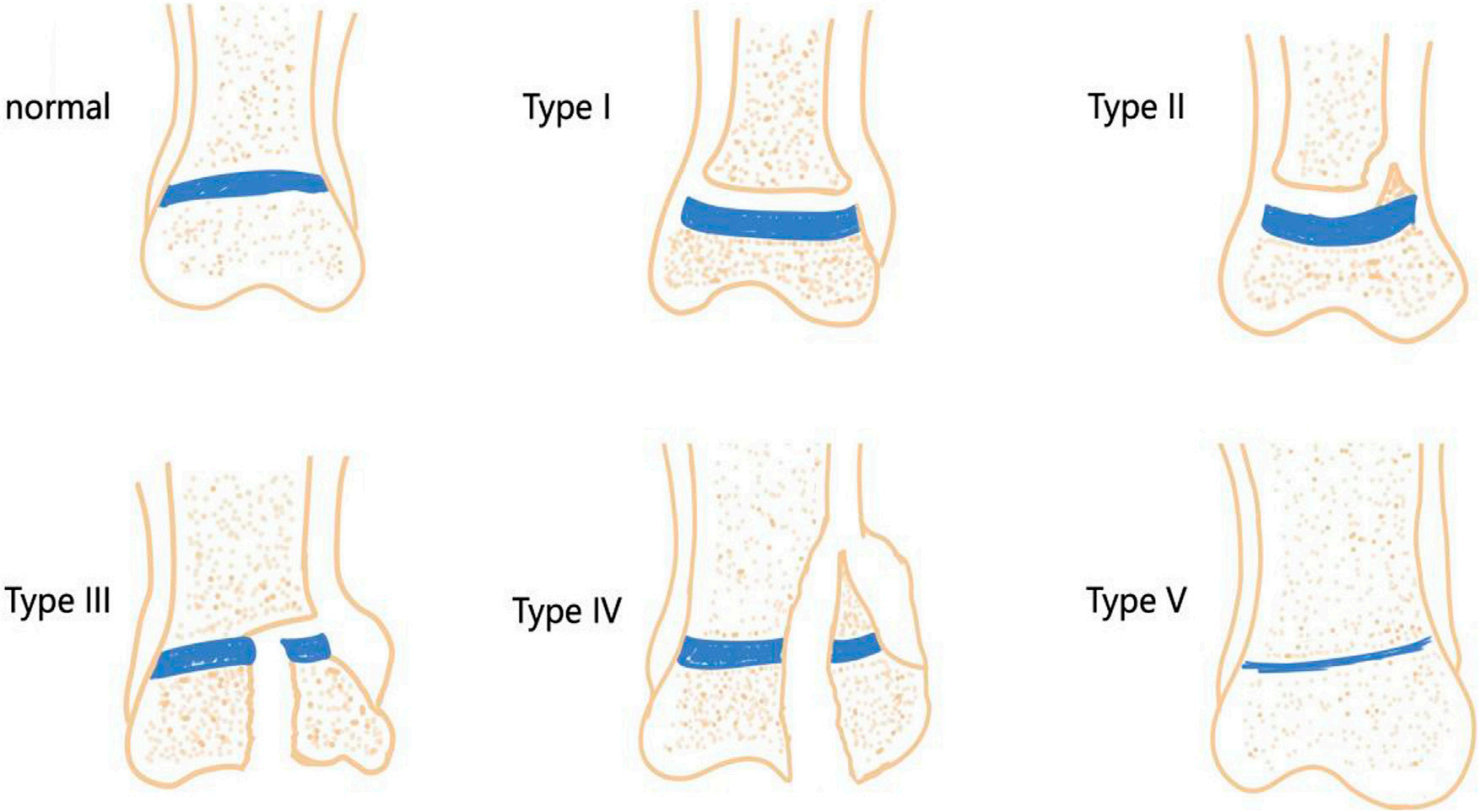
Type of growth plate injury.

The growth plate, a region of cartilage, is relatively fragile in children’s bones and can be easily damaged by infections, fractures, bone tumors, and iatrogenic injuries. The most common sites of growth plate injuries include the ankle, distal femur, and distal radius (MacIntyre and Dewan, 2016). Epidemiological studies show that growth plate injuries account for 15%–30% of all skeletal injuries in children (Eid and Hafez, 2002). The Salter-Harris (SH) system is the most widely used method for classifying growth plate injuries in clinical practice. The system is divided into five types based on injury location and prognosis (Chen et al., 2020) (Figure 1): Type I injuries involve a fracture across the entire growth plate, with an incidence rate of approximately 5%; Type II injuries involve an oblique fracture that spans the growth plate and penetrates the metaphysis, making them the most common type, accounting for approximately 75%; Type III injuries involve a fracture surface that passes through the growth plate and obliquely enters the epiphysis, with an incidence rate of approximately 10%; Type IV injuries involve a longitudinal fracture surface that penetrates the growth plate from the articular surface, ultimately reaching the metaphysis of the long bones, with an incidence rate of approximately 10%; Type V injuries are the rarest and involve a compressive injury to the growth plate, with the highest likelihood of leading to bone bridge formation. In the SH system, types III, IV, and V primarily damage the upper part of the growth plate, which can disrupt its nutrient vessels, leading to growth arrest in long bones and the formation of bone bridges. Types I and II typically damage the lower section of the growth plate, resulting in less impact on its blood supply and a relatively better prognosis.

**FIGURE 2:**
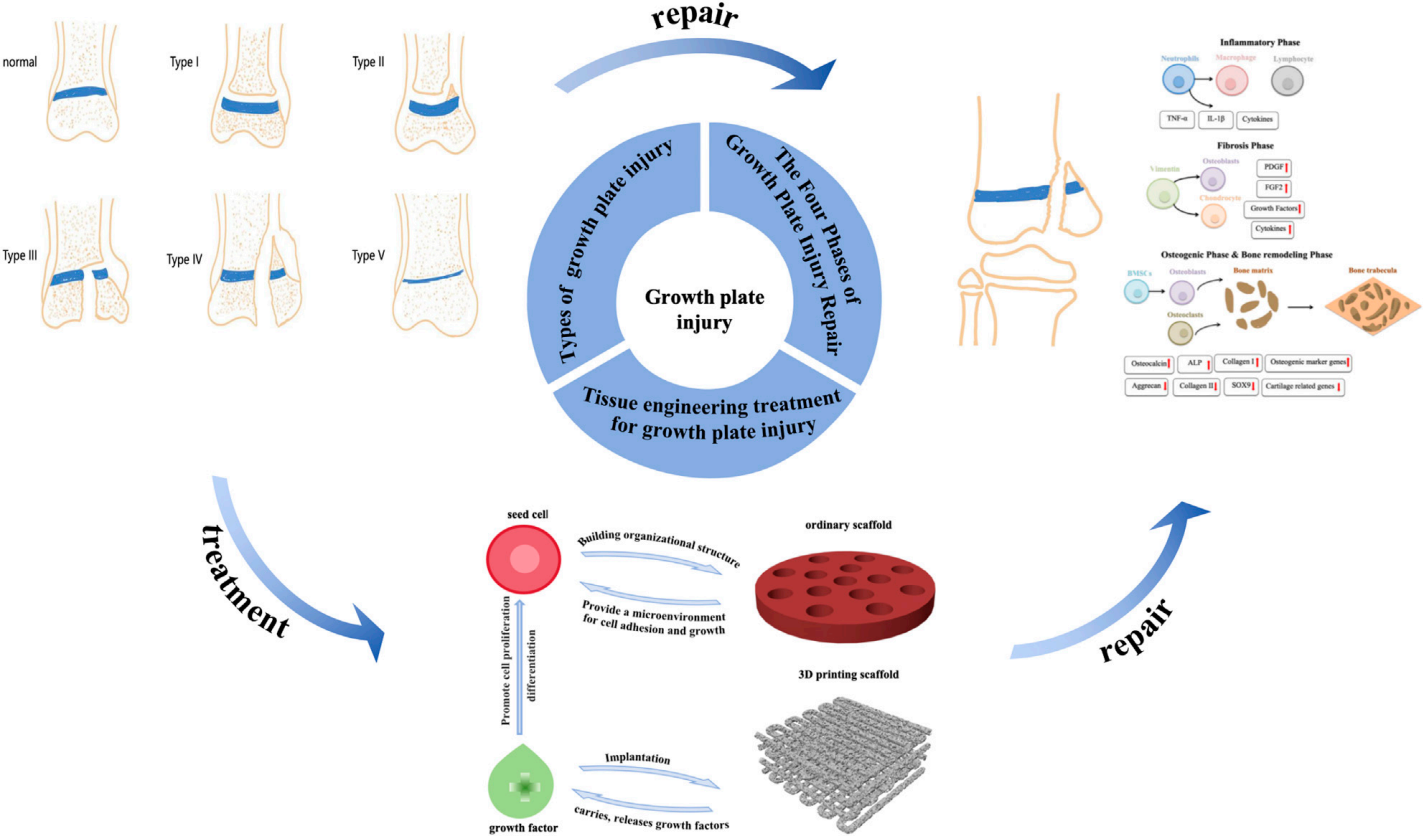
Growth plate injury and application of tissue engineering.

Due to the limited self-regenerative ability of cartilage, when growth plate is damaged, it is often replaced by undesired bone tissue, generating a bone bridge that obstructs normal limb growth and may result in length discrepancies and angular deformities (Gigante and Martinez, 2019). Current clinical treatment methods typically involve surgical resection of the bone bridge, followed using implantable materials, such as autologous fat or muscle, to fill the defect site. However, the poor integration of currently available implant materials with host tissues often results in subsequent complications, with a clinical success rate of less than 35%. Additionally, clinical treatment methods may lead to secondary injuries or bone bridge recurrence, ultimately resulting in a poor prognosis. Therefore, developing new methods to prevent bone bridge formation and promote growth plate tissue regeneration is essential. In recent years, with the advancement of cartilage repair theories, cartilage tissue engineering has emerged as a potential alternative treatment for growth plate injuries (Ladenhauf et al., 2020). Tissue engineering technology consists of three key components: seed cells, growth factors, and scaffolds. Seed cells are cultured *in vitro* and implanted into scaffolds to form cell-loaded scaffolds (Shaw et al., 2018). The *in situ* microenvironment influences seed cells post-implantation, making the addition of growth factors to the scaffold a crucial step in promoting cell differentiation. Furthermore, scaffolds used in tissue engineering are typically made from biocompatible and biodegradable materials, with specific methods used to form structures that possess three-dimensional integrity and mechanical strength. After implantation into growth plate defects, the scaffolds gradually degrade while supporting the formation of new cartilage tissue, serving as substitutes for growth plate defect fillers (Li et al., 2017). Despite promising results in many studies, there is currently no consensus on the optimal materials, seed cells, or growth factors (Erickson et al., 2018). As such, further research in tissue engineering is essential for future advancements. This article will systematically review the progress in tissue engineering treatments for growth plate injuries, highlighting both the challenges and prospects for clinical applications (Figure 2).

## 2 The four phases of growth plate injury repair

### 2.1 Inflammatory phase

**FIGURE 3:**
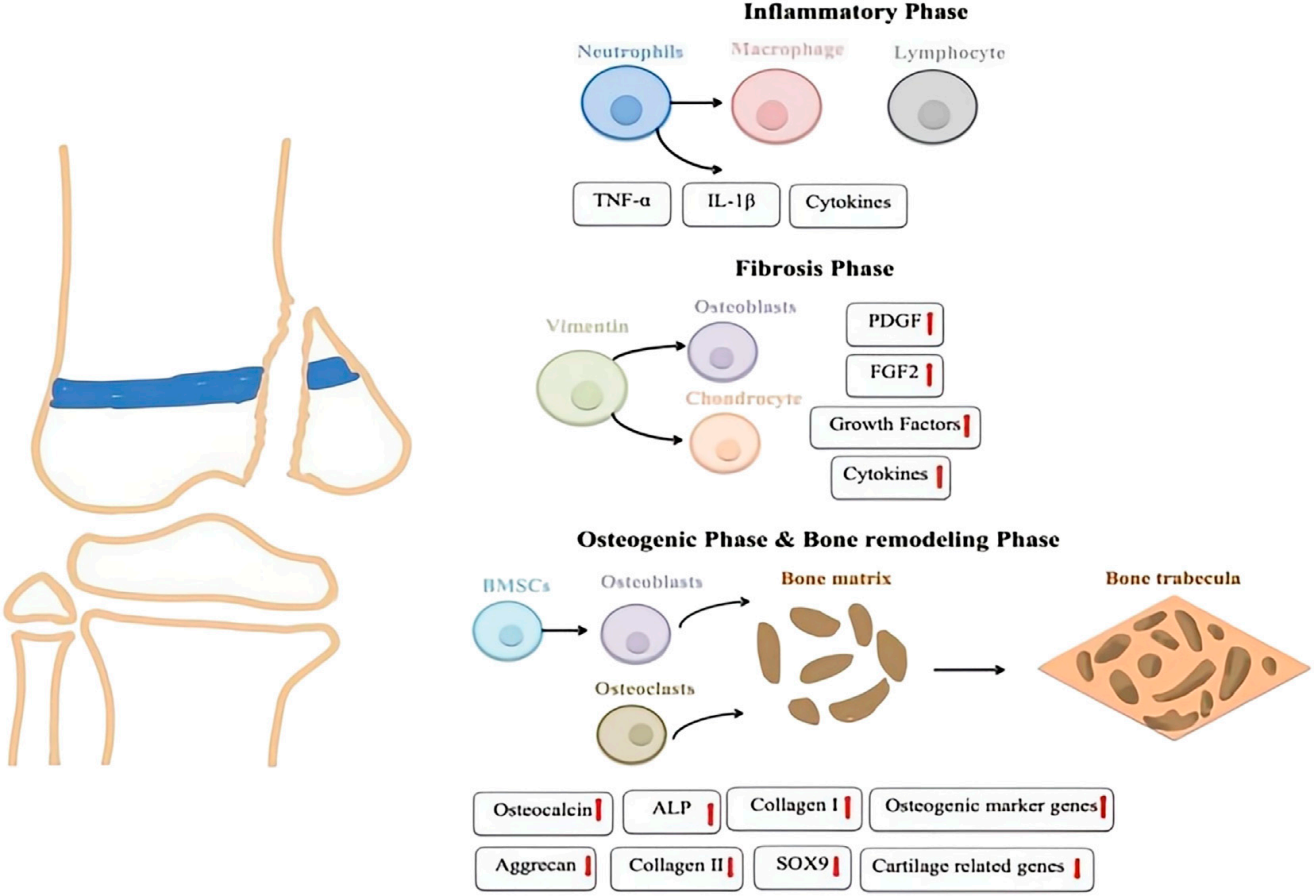
Four stages of Growth Plate Injury.

The first stage of growth plate injury repair is the inflammatory stage (Figure 3), marked by local reactions that recruit and activate numerous inflammatory cells. Following injury, the immune system responds quickly through complex mechanisms, promoting the infiltration of inflammatory cells to the site. In this process, upregulation of cytokines and mediators plays a crucial role. Levels of cytokines, such as tumor necrosis factor alpha (TNF-α) and interleukin-1 beta (IL-1β), are significantly elevated. These cytokines enhance the inflammatory response, regulate vascular permeability, increase blood flow, and facilitate the entry of immune cells and repair factors into the tissue (Zhou et al., 2006; Zhou et al., 2004). Cytokines like TNF-α and IL-1β activate downstream signaling pathways that promote the release of additional inflammatory factors, maintaining the local environment and supporting subsequent repair stages. A prior study examined the roles of two inflammatory mediators, cyclooxygenase-2 (COX-2) and inducible nitric oxide synthase (iNOS), in the injury response using a rat growth plate injury model. The findings indicated that COX-2 and iNOS mediate the inflammatory response triggered by the injury, potentially aiding the differentiation of mesenchymal cells into chondrocytes, and promoting bone remodeling during the repair process at the growth plate injury site (Arasapam et al., 2006).

### 2.2 Fibrosis phase

The fibrotic stage typically begins 3–7 days after injury, characterized by the accumulation of vimentin-positive mesenchymal stem cell (MSCs)s at the damaged site. These stem cells are crucial in the repair process, as they differentiate into osteoblasts and chondrocytes in response to appropriate stimulation. This differentiation is essential for the reconstruction and repair of bone and cartilage tissues. As the repair process progresses, these MSCs contribute to both the structural remodeling of the damaged areas and the cellular and signaling support necessary for efficient repair. Numerous studies have shown that the mRNA levels of fibroblast growth factor 2(FGF-2) and platelet-derived growth factor BB(PDGF-BB) are significantly upregulated during the fibrosis stage, suggesting that these two growth factors may play a crucial role in the repair process of growth plate injuries (Zhou et al., 2006; Zhou et al., 2004). FGF-2 is involved in the proliferation, migration, and differentiation of mesenchymal cells and bone progenitor cells (Farré et al., 2007). A recent study by Chung et al. demonstrated that inhibiting PDGF-R signaling during the fibrotic phase reduces the proliferation of mesenchymal cells and the formation of bone or cartilage tissues at the injury site, highlighting the essential role of PDGF in the fibrotic phase of growth plate repair (Chung et al., 2009).

### 2.3 Osteogenic phase

As mesenchymal cells differentiate into trabecular and cartilage tissues at the injury site, the repair process of growth plate injuries progresses into the osteogenic phase. During this osteogenic stage, MSCs gradually differentiate into osteoblasts, which begin secreting the bone matrix and form preliminary bone trabeculae. Concurrently, cartilage tissue also forms at the site of injury and gradually is replaced by bone tissue as the repair process advances, leading to the completion of bone reconstruction. During this osteogenic phase, the expression of osteogenic marker genes (such as osteocalcin, alkaline phosphatase (ALP), and collagen I) is upregulated (Erickson et al., 2018). In parallel, the expression of chondrogenic markers like Aggrecan, Collagen II, and the cartilage-specific transcription factor SOX9 gradually decreases (Chung et al., 2006). The overall change in gene expression marks the progression of the repair process from cartilage formation to true ossification, with bone tissue progressively replacing cartilage tissue to restore the functional integrity of the injured area.

### 2.4 Bone remodeling phase

Bone remodeling usually occurs several weeks to months after an injury, marking the final stage of bone repair. During this phase, osteoclasts resorb the irregular bone tissue formed in earlier stages, while osteoblasts re-establish the bone matrix as new bone forms (Heath et al., 1991). Bone marrow cells migrate to the injury site and settle within the newly formed bone marrow cavity, aiding the maturation and integration of new bone tissue. As the process progresses, the initially formed bone trabeculae undergo further modifications, gradually improving the stability and maturity of the bone structure. Over time, the function of the repaired area approaches normal levels, culminating in the complete restoration of both bone structure and function.

## 3 Tissue engineering technology

**FIGURE 4:**
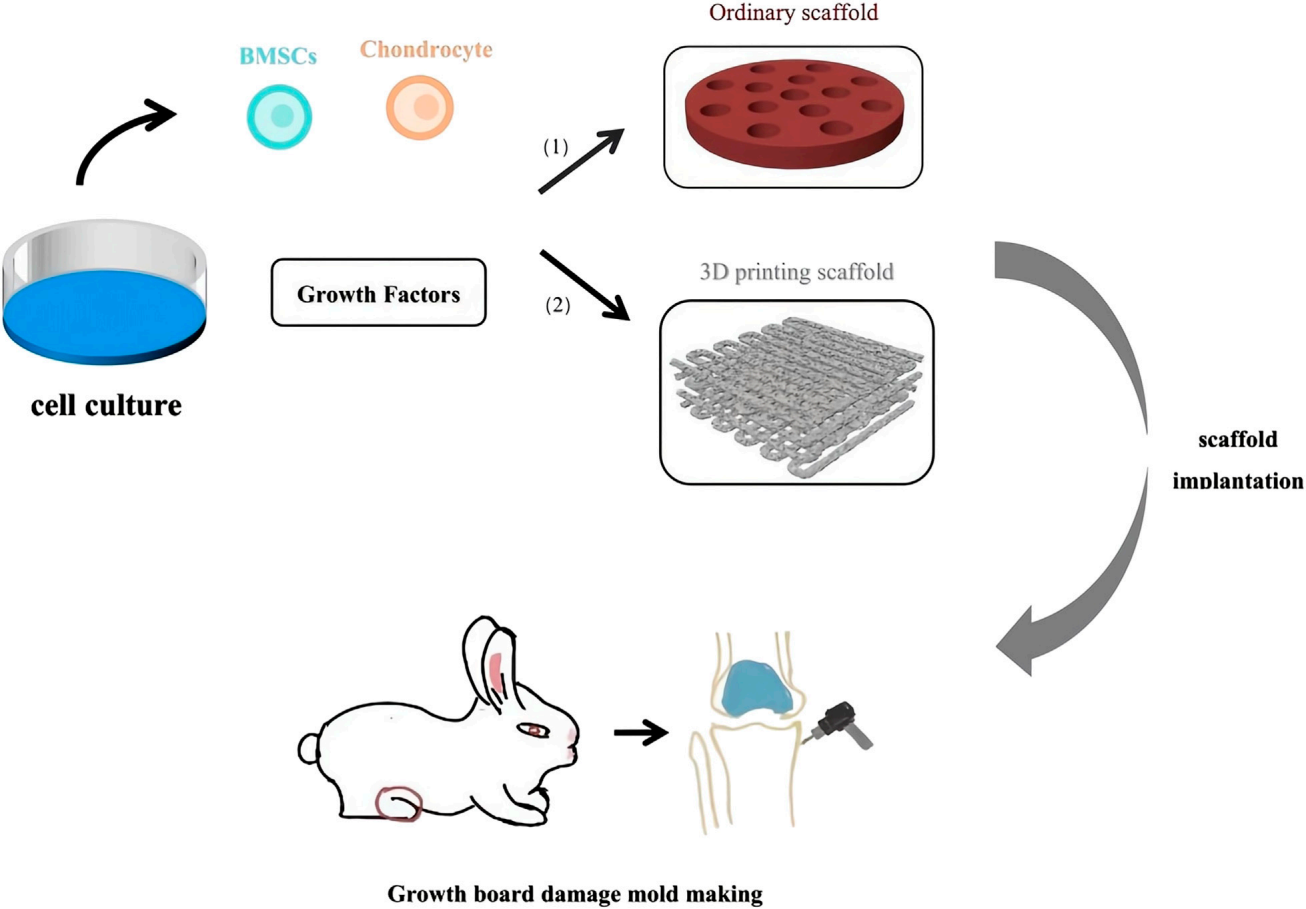
Tissue Engineering treatment for Growth Plate Injury. (1) Scaffold made of natural materials and composite materials. (2) Scaffold made by 3D printing technology.

Tissue engineering is an emerging field that has garnered significant attention in recent years. The core principle involves culturing and expanding normal tissue cells *in vitro*, which are then adsorbed onto a biocompatible biomaterial that is gradually absorbed by the body. This process results in the formation of a cell-biomaterial complex, which is then implanted into the site of tissue or organ damage. Over time, the biomaterial degrades and is absorbed by the body, while the cells grow, differentiate, and eventually form new tissues that closely resemble the original tissue’s morphology and function. This process facilitates effective wound repair and functional tissue reconstruction (Figure 4).

**FIGURE 5:**
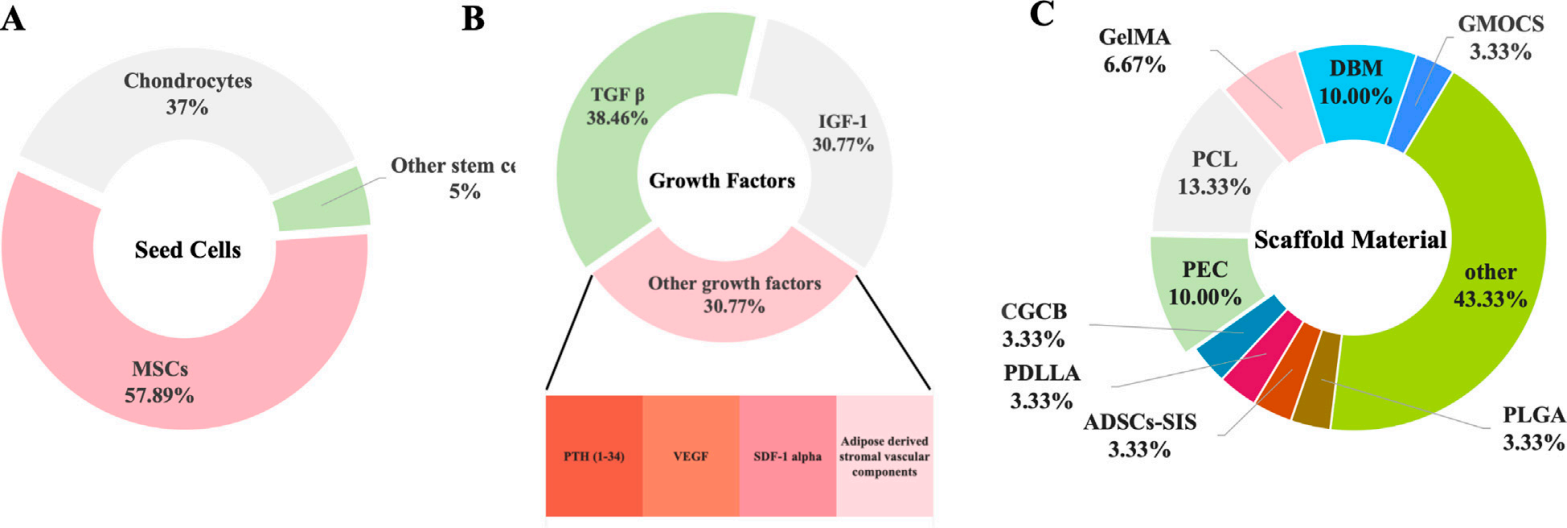
The Three Elements of Organizational Engineering. **(A)** The frequency and influence of the cells. **(B)** The frequency and influence of the growth factors. **(C)** The frequency and influence of the biomaterials.

Recent studies have demonstrated that tissue engineering techniques significantly aid in the repair of growth plate injuries (Table 1) (Figure 5). Methods such as seed cell therapy, scaffold material implantation, and the application of growth factors enable tissue engineering to significantly enhance the repair of growth plates. These approaches not only support the restoration of growth and development in the damaged area but also offer innovative solutions and new possibilities for clinical treatment.

**TABLE 1:** Multiple studies on Tissue Engineering treatment of Growth Plate Injury.

Author	Year	Seed cells	Growth factors	Scaffold material	Preparation technology	Experimental animal
Gültekin et al. (2020)	2020	MSCs and Chondrocytes		Cell thin layer	Cell culture	New Zealand White Rabbit
Wang et al. (2023)	2023	MSCs and Chondrocytes		PCL scaffold	3D printing technology	New Zealand White Rabbit
Erickson et al. (2020)	2020	MSCs and Chondrocytes		Alginate chitosan hydrogel	Freeze-Drying Technique	Rat
Fan et al. (2023)	2023	MSCs	PTH (1–34)	PCL scaffold	3D printing technology	New Zealand White Rabbit
Qiang et al. (2023)	2023	MSCs	VEGF, IGF-1	GelMA hydrogel	Water in oil in water emulsion technology	New Zealand White Rabbit
Yu (2019)	2019	MSCs	TGF β 3	Hydrogel	3D printing technology	Rabbit
Yu et al. (2022)	2022	MSCs	TGF β 3	PEG hydrogel	3D printing technology	Rabbit
Guan et al. (2022)	2022	MSCs and Chondrocytes		GMOCS hydrogel	Freeze-Drying Technique	Rat
Erickson et al. (2021)	2021	MSCs and Chondrocytes	SDF-1 alpha, TGF - β 3	Lotion free chitosan genipin micro gel	Freeze-Drying Technique	Rat
Sananta et al. (2020)	2022	ADSCs	Adipose derived stromal vascular components		SVF extraction technology	Rat
Lee et al. (2016)	2016	Chondrocytes		CTA	Cell culture	New Zealand White Rabbit
Li et al. (2021)	2021	MSCs		CS hydrogel/PCL hybrid	3D printing technology	Rabbit
Sundararaj et al. (2015)	2012		IGF-I	PLGA scaffold	Double emulsion method	Rabbit
Clark et al. (2015)	2015	MSCs	IGF-I	PLGA scaffold	Double emulsion method	New Zealand White Rabbit
Azarpira et al. (2015)	2015	MSCs		Chitosan	Cell culture	Albino rabbit
Tomaszewski et al. (2014)	2014	Chondrocytes			Cell culture	New Zealand White Rabbit
Planka et al. (2012)	2011	MSCs and Chondrocytes		Type I collagen/Chitosan	Nanotechnology	Miniature pig
Yoshida et al. (2012)	2011	MSCs			Cell culture	New Zealand White Rabbit
McCarty et al. (2010)	2020	MSCs	Transforming Growth Factor - β 1 (TGF β 1)	Gelatin sponge scaffold	Cell culture	Sheep
Plánka et al. (2011)	2011	MSCs and Chondrocytes		Type I collagen/Chitosan	Cell culture	Miniature pig
Li et al. (2017)	2017	MSCs		ECM	Cell culture	New Zealand White Rabbit
Jin et al. (2006)	2006	Chondrocytes		DBM scaffold	Cell culture	New Zealand White Rabbit
Li et al. (2004)	2004	MSCs		Chitin	Cell culture	Rabbit
Tobita et al. (2002)	2001	Chondrocytes		Unterminated collagen gel	Cell culture	Rabbit
Park et al. (1994)	1994	Chondrocytes			Cell culture	Newborn dog
Yang et al. (2002)	2002	Chondrocytes			Cell culture	New Zealand White Rabbit
Luo et al. (2003)	2003	Chondrocytes		DBM scaffold	Cell culture	Rabbit
Wang (2023)	2023	MSCs and Chondrocytes		PCL scaffold	3D printing technology	Rabbit
Yang et al. (2011)	2011	ADSCs		ADSCs-SIS	Cell culture	New Zealand White Rabbit
Cai (2004)	2004	MSCs		PDLLA	Cell culture	Japanese white Rabbit
Jiang et al. (2020)	2020	Chondrocytes		DBM scaffold	Decalcification technique	Rabbit
Coleman et al. (2013)	2013	MSCs	TGF β	agarose gel	Cell culture	Rat
Adhikari et al. (2024)	2024	MSCs		alginate/chitosan PEC hydrogels	Gene Delivery technique	Rat
Stager et al. (2022)		MSCs		alginate/chitosan PEC hydrogels	Cell culture	Rat
Guo et al. (2025)	2025	ATDC5		CGCB	Freeze-Drying Technique	Rat
Zhang et al. (2025)	2025	BMSCs	IGF-1	GelMA-DBNC-Alg hydrogels	Gelation	Rat

### 3.1 Seed cells

Skeletal stem cells (SSCs) are multipotent progenitors residing within the bone marrow that possess the capacity to differentiate into osteoblasts and chondrocytes. They play a critical role in regulating the local microenvironment by secreting various growth factors, thereby promoting the repair of bone tissue. Progenitor cells, positioned between stem cells and terminally differentiated cells, possess the ability to differentiate into specific chondrogenic and osteogenic progenitor cells, facilitating bone repair and restoring the normal function of the growth plate. Previous studies utilized the FoxA2CreERT2/+; Tomatofl/+ mouse model, wherein FoxA2+ cells were successfully labeled through tamoxifen treatment, enabling the tracking of their distribution at various time points within the growth plate and cartilage regions. The results indicated that FoxA2+ cells not only retain proliferative potential within the growth plate but also differentiate into both chondrocytes and osteoblasts (Muruganandan et al., 2022). Additionally, using the PTHrP-CreER mouse model, researchers manipulated PTHrP + cells in the growth plate by activating or deleting the PTHrP gene. The findings demonstrated that PTHrP + cells exhibit stem cell-like characteristics in the dormant zone of the growth plate and have the capacity to differentiate into various chondrocyte subtypes during injury repair. Further investigations using the PTHrP-CreER; R26R-Confetti mouse model revealed that individual PTHrP-CreER + dormant chondrocytes clonally produce multiple chondrocyte types, further confirming the pluripotency and stem cell properties of PTHrP + cells within the growth plate (Mizuhashi et al., 2018; Murphy et al., 2020). Seed cells are crucial in tissue engineering, especially for treating growth plate injuries. In two rabbit studies, researchers induced epiphyseal defects in the proximal growth plate to create a growth arrest model. Bone bridge resection was performed, followed by the separate implantation of agarose (Chen et al., 2003) with chondrocytes and agarose (Lee et al., 1998) with MSCs. This therapy corrected tibial angle deformation and stagnation in growth. Seed cell sources include autologous and allogeneic cells, along with stem cells like induced pluripotent stem cells (iPSCs), embryonic stem cells (ESCs), and adipose-derived stem cells (ADSCs). ADSCs combined with the small intestinal submucosal layer have been used to treat rabbit growth plate defects. The most widely used cells in tissue engineering for treating growth plate injuries are chondrocytes and MSCs.

#### 3.1.1 Mesenchymal stem cells

MSCs are a type of stem cell with multipotent differentiation potential, commonly found in tissues such as bone marrow, adipose tissue, the umbilical cord, dental pulp, and peripheral blood. These cells possess robust self-renewal capabilities and can differentiate into chondrocytes under appropriate conditions, making them key in cartilage tissue repair. As a result, MSCs are frequently used as seed cells in tissue engineering (Choi et al., 2018). For example, Cai (2004) combined bone marrow MSCs with the biological carrier, racemic Poly-D,L-lactic acid (PDLLA), to repair bone marrow defects in rabbits. The results demonstrated that tissue-engineered implants alleviated limb shortening and angular deformities following growth plate injury. Similarly, Li et al. (2004) utilized chitin as a scaffold material for periosteal MSCs, which were implanted into tibial defects in rabbits. This method effectively corrected length differences and angular deformities resulting from bone defects. Azarpira et al. (2015) established a growth plate injury model in the distal femur of albino rabbits and implanted a chitosan scaffold seeded with MSCs at the defect site. At higher cell concentrations, the chitosan-based MSC constructs effectively repaired epiphyseal defects. Furthermore, Li et al. (2017) employed freeze-drying technology to create oriented extracellular matrix (ECM) scaffolds, designed to mimic the structure and material properties of natural growth plates. After culturing bone marrow MSCs within these scaffolds and implanting them into rabbit defect models, the study found that the MSCs promoted chondrocyte regeneration and prevented bone bridge formation, reducing angular deformities and length differences in the rabbits.

#### 3.1.2 Chondrocytes

Chondrocytes are specialized cells that secrete ECM components, including collagen and proteoglycans, essential for cartilage structure and function. Cartilage cell therapy involves transplanting autologous or allogeneic chondrocytes to injured growth plate sites to stimulate regeneration and repair. In 1984, Park et al. (1994) transplanted autologous chondrocytes into the epiphyseal injury site of newborn dogs. The results showed that, compared to the control group, the tibial injury site exhibited significantly reduced angular deformity after chondrocyte transplantation, with the cartilage mass fusing with the surrounding growth plate. This study confirmed that chondrocyte transplantation effectively prevents bone bridge formation and promotes growth plate repair. The study also found that high-density cultured chondrocytes did not trigger immune rejection, attributed to the protective effect of the synthesized cartilage matrix, which shielded the chondrocytes’ antigenicity, preventing immune exposure (Chen et al., 2003; Lee et al., 1998). Tobita et al. (2002) compared autologous chondrocytes with autologous adipose tissue and found that chondrocytes, cultured in collagen-free gel, synthesized extracellular matrix over an extended period at the defect site. These chondrocytes survived during bone replacement, even after bone marrow cells invaded (Choi et al., 2018). The study showed that transplantation of autologous chondrocytes cultured in peptide-free collagen gel significantly reduced angular deformities and length discrepancies in the rabbit tibia, compared to autologous fat transplantation. Luo et al. (2003) transplanted autologous chondrocytes onto demineralized bone matrix (DBM). After 1 week of culture, they implanted the composite into the growth plate injury site on the inner side of the rabbit tibia. The results showed that timely implantation of autologous chondrocytes effectively prevented tibial deformities. Finally, Lee et al. developed a scaffold-free, tissue-engineered cartilage tissue analog (CTA) using suspended chondrocyte culture for transplantation into a rabbit growth arrest model. The study found that CTA transplantation minimized deformities in the rabbit growth plate injury model and promoted repair by reducing bone bridge formation.

Although chondrocyte therapy has been shown to play a critical role in repairing growth plate injuries by reducing surgical trauma, minimizing immune rejection, and promoting cartilage regeneration, it still faces significant challenges. These challenges include the limited proliferative capacity of chondrocytes and the poor long-term stability of regenerated cartilage. In contrast, MSCs exhibit strong proliferative potential, driving increased interest in combining MSCs with chondrocytes to treat growth plate injuries. Gültekin et al. (2020) co-implanted bone marrow-derived MSCs and chondrocytes into rabbit epiphyseal defects, observing successful regeneration of the growth plate at the defect site. Research indicates that this combined therapy can effectively repair growth plate injuries; however, it generally requires biodegradable scaffolds as carriers to support and guide cell growth. These scaffolds enhance cell survival, proliferation, and accelerate the repair process. For instance, Planka et al. (2012), Plánka et al. (2011) used miniature pigs as experimental models, implanting allogeneic MSCs and chondrocytes into defect sites along with a novel scaffold made from type I collagen and chitosan nanofibers. The setup aimed to compare longitudinal bone growth and eversion angles of the left and right lower limbs. The results demonstrated that this scaffold effectively prevented growth disorders and angular deformities in the distal femoral epiphysis. Additionally, the MSCs and chondrocyte combination therapy, using alginate-chitosan scaffolds, prevents bone bridge formation and promotes cartilage regeneration in rats with epiphyseal injuries (Erickson et al., 2020).

### 3.2 Growth factors

Growth factors are proteins or hormones that regulate cell growth, differentiation, and repair, and are widely involved in the processes of growth, development, immune response, and healing. They can promote chondrocyte proliferation and induce their transformation into mature cartilage or bone cells; growth factors also promote angiogenesis and bone tissue reconstruction, regulate inflammatory responses, and play a crucial role in the repair of growth plate injuries. In treating growth plate injuries, various growth factors, including bone morphogenetic proteins (BMPs), transforming growth factor-β (TGF-β), vascular endothelial growth factor (VEGF), fibroblast growth factor (FGF), and insulin-like growth factors (IGFs), have been widely studied and shown to have good repair effects.

#### 3.2.1 Transforming growth factor-β (TGF-β)

TGF-β is a multifunctional cytokine involved in numerous biological processes, including cell proliferation, differentiation, migration, and extracellular matrix synthesis. It plays a critical role in regulating the proliferation, differentiation, and repair of cartilage and bone cells. In chondrocytes, TGF-β primarily influences extracellular matrix synthesis through the SMAD2/3 and MAPK signaling pathways, participates in the anti-inflammatory response of cartilage tissue (Thielen et al., 2019), and promotes the differentiation of MSCs into chondrocytes (Grafe et al., 2018). Coleman et al. (2013) demonstrated that adding TGF-β to the culture medium induced the expression of chondrogenic differentiation-related genes, including Sox-9, type II collagen, and proteoglycans, in MSCs under *in vitro* conditions. The results showed a significant increase in the expression of these genes, suggesting that TGF-β effectively promotes chondrogenic differentiation and exhibits strong cartilage-inducting potential *in vitro*. However, in a study by McCarty et al., autologous bone marrow-derived MSCs were seeded onto a gelatin sponge scaffold containing TGF-β1 and transplanted into a surgically created defect in the proximal growth plate of sheep tibias. Five weeks after surgery, examination of the implant revealed that while autologous MSCs did not form new cartilage at the defect site, they did promote the formation of dense fibrous tissue and inhibited bone bridge formation (McCarty et al., 2010).

#### 3.2.2 Insulin-like growth factor I (IGF-I)

IGF-I is a peptide hormone primarily synthesized by the liver and other tissues. Its structure is similar to insulin, which is why it is termed “insulin-like.” IGF-I activates the downstream PI3K/Akt and MAPK signaling pathways upon binding to its receptors, promoting chondrocyte proliferation and differentiation, stimulating cartilage matrix formation, and inhibiting excessive ossification and abnormal fibrosis. Additionally, IGF-I facilitates the generation of new blood vessels, enhancing blood supply and supporting tissue repair and regeneration. To explore the therapeutic potential of IGF-I in growth plate injury, Sundararaj et al. (2015) developed an *in vivo* tissue engineering approach, delivering IGF-I locally via a cell-free polylactic acid-glycolic acid copolymer (PLGA) scaffold. *In vitro*, bone marrow mesenchymal stem cells (BMCs) were seeded onto PLGA scaffolds and cultured for 3 weeks. Compared to the control group, IGF-I-loaded scaffolds significantly accelerated cell proliferation and increased glycosaminoglycan (GAGs) content. *In vivo*, PLGA scaffolds were transplanted into the growth plate defect site of rabbit tibias, where thin cartilage tissue bands were observed at the injury site under HE staining. Both *in vitro* and *in vivo* experiments showed that IGF-I-loaded PLGA scaffolds facilitated new cartilage formation at the injury site, promoting growth plate repair. Building on this work, Clark et al. (2015) conducted a follow-up study introducing a new experimental group with IGF-I-loaded PLGA scaffolds containing BMCs. The results indicated that both types of IGF-I-loaded scaffolds significantly increased the density of hypertrophic chondrocytes. However, the scaffolds inoculated with cells showed the most pronounced proliferative effect on chondrocyte population formation. These findings suggest that IGF-I may induce the differentiation of bone marrow cells toward cartilage formation by promoting cell proliferation and enhancing the expression of chondrocyte markers. In a separate study, Bevacizumab, a monoclonal antibody against VEGF, was explored as a potential tool to reduce fibrosis or abnormal ossification. By inhibiting VEGF binding to its receptor VEGFR, Bevacizumab prevents abnormal blood vessel formation and may help reduce undesirable ossification. Qiang et al. (2023) developed a bilayer drug-loaded microsphere system, with the outer layer containing Bevacizumab and the inner layer containing IGF-I. These microspheres were combined with BMSCs and mixed with a gelatin methacryloyl (GelMA) hydrogel to form a composite hydrogel. The hydrogel, exhibiting excellent injectability and biocompatibility, was injected into a rabbit tibial injury model. The outer layer of the microspheres initially released Bevacizumab, inhibiting early osteogenic differentiation and preventing bone bridge formation, while the IGF-I in the inner layer was slowly released to promote cartilage differentiation and regeneration of growth plate cartilage.

#### 3.2.3 Parathyroid hormone 1–34 [PTH (1–34)]

PTH (1–34) is a fragment of PTH secreted by the parathyroid glands, primarily responsible for regulating calcium levels in the blood. PTH (1–34) activates downstream signaling pathways, such as the cAMP/PKA pathway, by binding to PTH receptors. This promotes the proliferation and differentiation of chondrocytes and BMSCs, enhances the calcification of chondrocytes, and accelerates bone growth and the repair of growth plate injuries. Fan et al. (2023) employed 3D printing technology to load PTH (1–34) into PLGA microspheres, which were then mixed with BMSCs and GelMA to create a scaffold for implantation into a rabbit model. The results indicated that, compared to the experimental group without PTH (1–34) loading, the PTH (1–34)/PLGA/BMSCs/GelMA PCL scaffold group demonstrated a significantly greater effect on cartilage formation.

#### 3.2.4 Other growth factors

Growth hormone (GH) plays a crucial role in regulating the chondrocytes of the growth plate. GH promotes skeletal growth by stimulating growth plate chondrocytes to secrete IGF-I (Locatelli and Bianchi, 2014). Additionally, GH directly enhances longitudinal bone growth (Ohlsson et al., 1998). Angiogenesis at the site of growth plate injury is a critical step in the conversion of fibrous repair tissue to bone tissue, with VEGF acting as a key regulator of angiogenesis. VEGF not only plays a central role in angiogenesis but is also indispensable in osteogenesis and bone remodeling. Rose et al. conducted anti-VEGF treatment in a rat tibial growth plate injury model and found that systemic VEGF blockade resulted in decreased blood flow and a significant reduction in bone repair tissue formation. This study further confirmed the essential role of VEGF-induced angiogenesis in bone formation, endochondral ossification, and the transformation of hypertrophic cartilage to trabecular bone at the site of growth plate injury (Chung et al., 2014).

BMPs play a significant role in bone development and repair, particularly in osteoblast differentiation and cartilage formation (Leboy, 2006; Tsuji et al., 2006). Early studies utilizing microarray gene expression analysis revealed that BMP signaling pathways were upregulated at the injury site during the repair process in a rat growth plate injury model (Su et al., 2021). Further research by Su et al. demonstrated that BMP signaling activation was markedly increased in both the injured growth plate and adjacent regions. Inhibition of BMP signaling significantly impaired bone repair at the site of growth plate injury and reduced the hypertrophic degeneration induced by the injury in the adjacent growth plate region.

### 3.3 Scaffold material

A scaffold is a three-dimensional support structure in tissue engineering, designed to provide space for cell growth, expansion, and differentiation, while preserving the morphology and function of the engineered tissue. For growth plate repair, the scaffold material must meet the following requirements: good biocompatibility and biodegradability, appropriate mechanical strength, and the ability to promote cell adhesion and proliferation. Common scaffold materials include natural materials such as collagen (Planka et al., 2012; Plánka et al., 2011), chitosan (Azarpira et al., 2015; Li et al., 2004), ECM (Li et al., 2017), decalcified bone matrix (Jin et al., 2006), and others. Synthetic materials include PLGA (Sundararaj et al., 2015; Clark et al., 2015), Polycaprolactone (PCL) (Wang et al., 2023; Fan et al., 2023; Wang, 2023), polyethylene glycol (PEG) (Stager et al., 2022), PDLLA (Cai, 2004). Composite materials include chitosan (CS) hydrogel/PCL hybrids (Lee et al., 2016), which combine natural and synthetic materials to leverage their respective advantages.

PCL is a semi-crystalline aliphatic polyester commonly used in tissue engineering. Owing to its favorable hydrophobicity, excellent biocompatibility, and thermoplasticity, PCL has become one of the most used materials in 3D printing (Basturkmen et al., 2022; Bachtiar et al., 2021). Furthermore, PCL is biodegradable and can be combined with various drugs and growth factors. Research has shown that its higher molecular weight is closely associated with its slow degradation characteristics. Due to these advantages, PCL is widely considered an ideal biomaterial for preparing scaffolds in tissue engineering (Lee et al., 2016).

PEG hydrogel scaffolds are widely used materials in biomedicine. In tissue engineering, PEG hydrogel can be combined with chondroitin sulfate (ChS), cell adhesion peptides (RGD), and other extracellular matrix analogs, as well as TGFβ3, to construct a rigid 3D-printed structure. This composite material plays a crucial role in promoting the regeneration of growth plate cartilage tissue and the recovery of bone development (Bachtiar et al., 2021). Wang et al. developed a PEC hydrogel composed of alginate and chitosan, optimizing its hardness and mechanical properties by adjusting the polymer content. This hydrogel was designed as an injectable biomaterial system for the repair of growth plate injuries (Yu et al., 2022).

PLGA is a biodegradable and biocompatible polymer widely utilized for the delivery of growth factors, anti-inflammatory agents, and stem cells to modulate the local microenvironment, promote cartilage regeneration, and inhibit osseous bridge formation (Sundararaj et al., 2015; Shive and Anderson, 1997). Owing to its excellent processability, PLGA is compatible with advanced fabrication techniques such as 3D printing and electrospinning, enabling minimally invasive injection and site-specific therapies. However, its application in growth plate repair remains constrained by several limitations, including the acidity of its degradation byproducts, which can trigger local inflammation, as well as insufficient mechanical strength to withstand biomechanical stress in load-bearing regions (Fu et al., 2000; Asaw et al., 2012). Furthermore, PLGA alone exhibits suboptimal bioactivity for promoting cell adhesion and differentiation. To address these challenges, recent studies have explored composite strategies, such as blending PLGA with PCL to enhance mechanical performance, or incorporating bioactive molecules like PTH (1–34) and IGF-1 for controlled release, thereby providing sustained biochemical stimulation and improving cellular adhesion, proliferation, and cartilage regeneration (Fan et al., 2023).

Black Phosphorus (BP) is a two-dimensional material with excellent biocompatibility, making it suitable as a scaffold for cell growth and capable of regulating the release of growth factors, thereby playing a significant role in the repair of growth plate injuries. Guo et al. constructed a Curdlan/β-glycerophosphate/Chitosan gel containing Black Phosphorus (CGCB) (Guo et al., 2025). *In vitro* and *in vivo* experiments revealed that CGCB promoted the differentiation and migration of chondrocytes, effectively inhibited the formation of bony bridges, and facilitated the repair of growth plate injuries.

Considering the physiological structure and function of the growth plate, Zhuang et al. innovatively constructed a high-toughness, adaptive, double-crosslinked hydrogel (GelMA-DBNC-Alg hydrogels) through Schiff base bonding between GelMA and aldehyde-modified bacterial cellulose (DBNC), as well as ionic electrostatic interactions between sodium alginate (Alg) (Zhang et al., 2025). Additionally, IGF-1 growth factor was incorporated into the hydrogel to create a hypoxic microenvironment that activates IGF-1-related signaling pathways, promoting growth plate cartilage regeneration and minimizing the formation of bony bridges, thus providing an effective solution for the repair of growth plate injuries.

### 3.4 Preparation technology

Tissue engineering preparation technologies play a crucial role in the treatment of growth plate injuries. With the continuous development of tissue engineering, the precise design of scaffolds, selection of cell sources, and controlled release of bioactive molecules have provided new therapeutic strategies for growth plate repair. 3D printing and cell culture technologies enable the precise construction of three-dimensional scaffolds and the provision of suitable microenvironments for cell proliferation and differentiation. Meanwhile, water-in-oil (w/o) emulsification and double emulsification methods effectively deliver drugs and precisely control the release of growth factors, optimizing the repair process. Gene delivery technology introduces genes encoding growth factors, osteogenic factors, or differentiation regulators to the injury site via viral or non-viral vectors. These genes stimulate the differentiation of cells into osteoblasts or chondrocytes, promoting growth plate repair. Nanotechnology and freeze-drying techniques further enhance the biocompatibility and stability of scaffolds and cells, improving the repair outcomes. Combining SVF extraction technology provides additional stem cell sources, facilitating tissue regeneration and reconstruction.

#### 3.4.1 3D printing technology

The application of 3D printing technology in growth plate injury repair has increasingly focused on the precise modulation of the local microenvironment. By tailoring scaffold architecture, porosity, cell density, and spatial distribution of bioactive factors, 3D printing enables the recreation of the native structure and functional microenvironment of the growth plate, thereby directing cellular behaviors and promoting tissue regeneration (Velioglu et al., 2019). Studies have demonstrated that pore size plays a critical role in regulating chondrocyte proliferation and differentiation: smaller pores (90–250 μm) favor cell proliferation and maintenance of the chondrogenic phenotype, whereas larger pores (>400 μm) are associated with increased chondrocyte hypertrophy and apoptosis (Li et al., 2021; Wang, 2023; Han et al., 2021). These effects may be attributed to differences in local oxygen tension and nutrient availability. Notably, hypoxic conditions have been shown to enhance chondrocyte activity, while excessive nutrient supply may accelerate cellular senescence (Di Luca et al., 2016; Tanaka et al., 2022). Based on these findings, gradient scaffold designs that mimic the zonal architecture of the growth plate have emerged, allowing dynamic regulation of the local microenvironment. Moreover, the incorporation of composite biomaterials has expanded the functional capabilities of scaffolds. For example, 3D-printed PCL-GelMA scaffolds provide mechanical support, while embedded PLGA microspheres enable sustained release of PTH (1–34), closely mimicking the spatiotemporal biological processes involved in growth plate regeneration (Fan et al., 2023). Tidemark is a thin film (Chen et al., 2011) located between the cartilage layer and the calcified cartilage layer. During the development of the growth plate, the formation of tidemark marks the transition from cartilage to bone and plays a crucial role in regulating the calcification process of cartilage tissue, maintaining the balance between cartilage and bone (Gupta et al., 2005; Yildirim et al., 2023). Additionally, the presence of tidemark helps maintain the flexibility of the cartilage layer and joint mobility, thereby promoting the stability of the growth plate (Lyons et al., 2006). Previous studies have shown that the thickness and structural changes of the tidemark are closely related to the therapeutic outcomes of cartilage injuries and osteoarthritis (Deng et al., 2019). Khademhosseini et al. combined 3D printing technology with electrospun PCL membranes to design a continuous gradient biomaterial scaffold that simulates tidemark function, more effectively supporting cartilage repair (Effanga et al., 2024). Wang et al. (2023) used 3D printing to create bi-layered scaffolds with varying pore sizes to simulate growth plate regions. The results showed superior repair effects in promoting cartilaginous tissue formation. Similarly, Fan et al. (2023) fabricated PCL-GelMA scaffolds integrated with PTH 1–34 microspheres and BMSCs, which, when implanted into a rabbit growth plate injury model, significantly promoted new cartilage formation without deformities. These studies highlight the potential of 3D printing in enhancing cartilage differentiation and promoting regeneration.

Although experimental studies have demonstrated the significant potential of 3D printing technology in the tissue engineering treatment of growth plate injuries, several limitations remain. First, material selection and biocompatibility concerns impact the stability and efficacy of tissue repair by scaffolds, with the degradation rates of certain materials not aligning with the repair requirements of the growth plate (Adhikari et al., 2024). Second, current 3D printing technologies face challenges in directly printing complex vascular networks. While studies have explored promoting angiogenesis through the incorporation of vascularization channels or growth factors, such approaches continue to encounter technical and cost barriers in clinical applications (Alshaer et al., 2021). Additionally, 3D printing struggles with the accurate reconstruction of the growth plate’s complex multi-layered structure, and there remains a disparity between the mechanical properties of existing scaffolds and the biomechanical demands of the growth plate (Hann et al., 2021). While 3D printed scaffolds have shown promising repair outcomes in animal models, their long-term clinical efficacy remains uncertain, and issues regarding scaffold stability *in vivo* and the safety of degradation products require further investigation and validation.

#### 3.4.2 Other preparation technology

Electrospinning is an advanced fabrication technique that utilizes electrostatic forces to stretch polymer solutions or melts into ultrafine fibers, enabling the formation of fiber networks with diameters ranging from the nanometer to micrometer scale. These networks closely mimic the structure of the ECM. In this study, twin-screw extrusion combined with electrospinning was employed to fabricate functionally graded scaffolds with precisely controlled porosity and compositional gradients. This approach aims to replicate the spatial distribution of mineral and organic components in native bone tissue, thereby enhancing cell adhesion, proliferation, and subsequent bone regeneration (Erisken et al., 2011; Erisken et al., 2008). Despite its advantages, electrospinning suffers from several limitations, including low production efficiency, variability in fiber diameter, sensitivity to environmental conditions, and relatively high cost.

Freeze-drying is a dehydration process in which materials are frozen at low temperatures, followed by the sublimation of water directly from the solid to the vapor phase under vacuum. This method effectively preserves the porous architecture and porosity of bone scaffolds, maintaining their structural integrity while facilitating cell adhesion and proliferation, ultimately promoting new bone formation (Singh et al., 2022). However, freeze-drying also has inherent drawbacks, such as a complex processing procedure and the potential for structural damage during fabrication.

Small interfering RNA (siRNA) is an emerging RNA interference technology that can target and silence specific genes associated with the repair of growth plate injuries, modulating key signaling pathways such as the MAPK pathway that affect growth plate function, thereby promoting cartilage regeneration and inhibiting abnormal bone formation (Metz et al., 2020; Hu et al., 2020). Adhikari et al. used an alginate-chitosan PEC hydrogel to deliver siRNA therapeutics to BMSCs, targeting and inhibiting the p38-MAPK pathway within BMSCs to suppress osteogenic differentiation, thereby preventing ectopic bone formation and promoting cartilage regeneration in growth plate injury repair (Adhikari et al., 2024).

## 4 Conclusion

The main consequence of growth plate injury is disruption of normal bone growth, especially in the longitudinal growth of long bones like the femur and tibia. Traditional surgical treatments, including bone bridge resection and osteotomy, are widely used in clinical practice. Bone bridge resection effectively addresses limb growth arrest and angular deformities resulting from partial premature closure of the growth plate, particularly in patients with epiphyseal closure. However, this technique requires precision and carries a risk of recurrence, especially in patients with residual growth plate function (Adhikari et al., 2024). Osteotomy can correct angular deformities and limb asymmetry, but it is associated with high recurrence rates and limited efficacy in children nearing skeletal maturity (Badina et al., 2013). Minimally invasive techniques, such as arthroscopically assisted repair, involve smaller incisions, reducing tissue damage, promoting faster recovery, and minimizing complications (Fu et al., 2019). Intradermal fixation minimizes soft tissue damage and reduces complications, including infection and hematoma, particularly in cases involving open growth plates. This approach effectively repairs fractures, preserves normal bone growth, and reduces the risk of malunion (Isaka et al., 2021; Garrett et al., 2011). However, both techniques are technically demanding and require high skill levels.

Recent advances in tissue engineering present promising alternatives. Cells, scaffolds, and growth factors can be used to create structures that support tissue function and offer personalized treatment for growth plate injuries. Cartilage tissue engineering scaffolds typically include seed cells, growth factors, and scaffold materials. Common seed cells include MSCs and chondrocytes. MSCs can proliferate and differentiate into osteoblasts and chondrocytes, promoting bone and cartilage regeneration. They also secrete growth factors, such as VEGF and MMPs, enhancing tissue repair. However, issues with non-directed differentiation and unclear long-term effects of MSCs necessitate further investigation.

Cartilage cell therapy, with low immune rejection risk, can treat varying degrees of injury, but its complexity and cost remain challenging. Other stem cells, such as those from adipose tissue or umbilical cord blood, show promise for growth plate repair. Research into these cells’ characteristics and roles may lead to safer and more effective treatments. Growth factor therapy, which regulates cell processes such as proliferation, differentiation, and angiogenesis, also supports tissue repair. Key growth factors are BMPs, VEGF, TGF-β, and IGFs.

Effective tissue engineering for growth plate injuries depends on scaffolds that provide mechanical support and 3D structures. Ideal scaffolds should have good biocompatibility, mechanical strength, controllable degradation, and promote angiogenesis. Natural scaffolds, like collagen and gelatin, support cell adhesion and tissue regeneration but have low mechanical strength and limited availability. Synthetic scaffolds offer better control and mechanical strength but have lower biocompatibility. Recent studies combine natural and synthetic materials to enhance bone repair and reduce surgical risks.

3D printing technology has emerged as a key tool in scaffold design, enabling the creation of customized, precise scaffolds tailored to individual patient needs. This technology enhances tissue repair by promoting cell growth and improving nutrient exchange, leading to more effective and personalized treatment plans.

Tissue engineering has demonstrated significant potential for treating growth plate injuries. Its primary advantages include the use of biodegradable materials, which effectively minimize foreign body reactions and the need for secondary surgeries, as well as offering good individual adaptability and a broad range of applications. Compared to traditional treatment methods, tissue engineering approaches can significantly reduce the risks of complications and recurrence. However, the clinical application of this technology still faces numerous challenges. First, the high complexity of the growth plate’s structure and function makes it difficult for current technologies to fully reconstruct its three-dimensional architecture and physiological functions. Second, the availability of chondrocytes is limited, and their *in vitro* expansion potential is insufficient. While stem cells possess differentiation potential, their use is associated with the risk of ossification. Additionally, the mechanical properties, biocompatibility, and degradation rates of scaffold materials are still inadequate to meet the regenerative needs of growth plates. The delivery systems for growth factors also suffer from instability in controlled release, and significant improvements are needed to address individual variability in treatment outcomes. Furthermore, the high technological barriers, substantial costs, and strict regulatory requirements continue to hinder clinical translation. Nevertheless, the future holds considerable promise. By optimizing the properties of biomaterials, more accurate simulations of the growth plate’s physiological microenvironment can be achieved. Efficient techniques for targeted stem cell induction, which promote stable differentiation into chondrocytes, must also be developed. Furthermore, a precise and controllable bioactive factor delivery system should be established. By combining cutting-edge technologies such as 3D printing and gene therapy, personalized and functional treatment strategies can be achieved, accelerating the clinical application process and ultimately achieving effective regeneration and repair of growth plate injuries.
